# Supplementary material for: Sexual function index adaptation for breast cancer patients (FSFI-BC)- translation and psychometric properties of Persian version
Source: BMC Womens Health. 2024 Jan 24;24:66. doi: 10.1186/s12905-023-02836-9 (PMC10809462; doi:10.1186/s12905-023-02836-9)
Supplement: Supplementary file 1 — Supplementary Material 1 [file 12905_2023_2836_MOESM1_ESM.docx]

Title page

**Manuscript Title:**

**Sexual function index adaptation for breast cancer patients (FSFI-BC)- Translation and Psychometric properties of Persian version**

**Authors’ information:**

**Authors’ Full Name(s) and order of author(s):**

1. Marzieh Masjoudi
2. Zohre Keshavarz
3. Mohammad Esmaeil Akbari
4. Farah Lotfi Kashani
5. Malihe Nasiri
6. Hamid-Reza Mirzaei

**Authors’ affiliation:**

1- Assistant professor of Reproductive health, midwifery department, Islamic Azad University , Rasht Branch, Rasht, Iran

2- Associate professor of Reproductive health, Midwifery department, Shahid Beheshti University of Medical sciences, Tehran, Iran

3- Professor of Surgical Oncology, Cancer Research Center, Shahid Beheshti University of Medical Sciences, Tehran, Iran

4- Professor of Psychology, Islamic Azad University, Roudehen branch, Tehran, Iran

5- Assistant professor of biostatistics, basic science department, Shahid Beheshti University of medical sciences, Tehran, Iran

6- Professor of Radiation Oncology, Department of Radiotherapy, School of Medicine Shohada-e-Tajrish Hospital Shahid Beheshti University of Medical Sciences, Tehran, Iran

**Corresponding Author**:

Name: Zohre Keshavarz,

Affiliation: Associate professor of Reproductive health, Midwifery department, Shahid Beheshti University of Medical sciences ORCID ID: 0000-0001-7176-2481

Phone**:** +989123907712 email: [keshavarzzohre@yahoo.com](mailto:keshavarzzohre@yahoo.com)

keshavarzzohre57@gmail.com

Address: Vali Asr Ave., Niayesh Cross Road,

Niayesh Complex,

Tehran, Iran

Zip code: 1985717443

**Sexual function index adaptation for breast cancer patients (FSFI-BC)- Translation and Psychometric properties of Persian version**

**Abstract:**

**Background-** Effective interventions to improve sexual dysfunction in breast cancer survivors need screening of these dysfunctions with a suitable instrument. The aim of present study was translation and identifying psychometric properties of Female Sexual Function Index – Adapted for Breast Cancer (FSFI-BC) which has been specifically developed for breast cancer survivors.

**Method-** This methodological study was performed between February 2017 and October 2018. 200 breast cancer survivors in stage 1 or 2 who were selected through convenience sampling method, completed the questionnaire. Reliability was assessed by Cronbach’s alfa and test re-test analysis and construct validity was performed through confirmatory (CFA) and exploratory factor analysis( EFA).

**Results-**Six factors were extracted in exploratory factor analysis (EFA). These factors explained 74.6% of the total variance in in NSA group and 0.821 in SA group. Reliability evaluation indicated high internal consistency and good test re-test reliability. Cronbach's alpha coefficient in all areas of the tool was above 0.7 (the lowest and the highest measures were 0.885 and 0.945, respectively), which is a good indicator for reliability of an instrument. Confirmatory factor analysis showed an acceptable fitness for seven factors of FSFI-BC questionnaire (Normed Fit Index or NFI= 0.9 for both groups, Comparative of Fit Index or CFI= 0.93 and 0.92 , χ 2/df= 1.68 and 1.71 for SA(Sexually Active) and NSA(No Sexually Active) individuals, respectively) .

**Conclusion-**Study findings suggest that Persian version of FSFI-BC is a suitable instrument for sexual dysfunction screening in breast cancer survivors.

**Key words:**

Breast cancer, Sexual dysfunction, , FSFI-BC, Psychometric properties

**Background**

Breast cancer is one of the most common cancers and the second leading cause of death from women cancers in the world[1, 2] . The incidence of this cancer has increased in the last four decades and Iranian women with breast cancer are relatively younger than their Western counterparts[3]. Studies showed, breast cancer patients in Iran are at least 10 years younger than their counterparts in developed countries[4, 5]. In one study , the mean age of Iranian women at the time of diagnosis was 49-46 years[6]. Therefore, due to the younger age of affected women in Iran, these people may have different experiences, including in the field of sexual function [7]. Sexual dysfunction is the most common long-term cancer problem that affects the health of women with breast cancer and can occur at any stage of diagnosis, throughout treatment and the years after treatment. Sexual dysfunction is just as problematic as breast cancer itself, if not more so[2, 8-11].The reported prevalence of sexual dysfunction following the diagnosis and treatment of cancer varies between 15 and 100% in different studies. Although it is necessary to check the quality of these studies [7, 8, 10-13]. In a study conducted by Harirchi et al. In Iran, the prevalence of sexual dysfunction before and after treatment was 52% and 84%, respectively. This is a sign of impaired sexual function following treatment for breast cancer. [7].

The mortality rate of breast cancer has decreased over the past 20 years. As a result, we will see more affected women who will live longer. Therefore, the quality of life in general and the sexuality as one of the important components of quality of life in particular, should be given more attention [14-17]. However, not enough attention is paid by the treatment team and patients' sexual problems are not investigated [3, 14, 18-20].

Breast cancer survivors are often not comfortable to talk about their sexual problems. Health care providers also have difficulty entering their patients sexuality worlds [21, 22]. During breast cancer treatment, addressing sexual problems is not a priority. Both patient and therapist avoid talking about sexual concerns due to embarrassment, lack of privacy, time, or required skills[23].Furthermore, in most Asian countries, including Iran, sexual issues are considered as taboo and talking about these issues is associated with feelings of shame and guilt. Health care providers are not exception, so there is not any communication about the sexual concerns between providers and patients[24]. In a study by Masjoudi et al., neglecting the sexual concerns of the survivors and the lack of teamwork among the service providers were two of the most important obstacles to talk about the sexual concerns of patients from the patient- provider communication perspective[25].

Due to the high prevalence of breast cancer in Iran and the long-term survival of patients experiencing side effects, it is necessary to measure and evaluate the accompanying sexual problems [26].Measuring sexual dysfunction after breast cancer diagnosis and treatment with a suitable instrument help the providers to choose effective interventions to address this problem[27]. So far, more than 30 different tools have been introduced to assess sexual function; Many studies have used the Female Sexual Function Index (FSFI) extensively to assess the sexual function of breast cancer survivors[28-31], but some researchers believe that the 19-item questionnaire assesses vaginal intercourse rather than sexual dysfunction[32].

Given the barriers in this area, it is essential to use a reliable and easy-to-use tool to diagnose patients' sexual problems. This tool should be able to assess side effects and treatment outcomes. Also, it should be aligned with the DSM-5(Diagnostic and Statistical Manual of Mental Disorders -version 5) and ICD-10 (the International Classification of Diseases version 10) criteria’s. These dimensions include desire for sexual activity, arousal / excitement, orgasm, pain, distress / dysfunction[33]. At the same time, the tool should be able to address the specific concerns of breast cancer survivors[14, 16].

FSFI is the most widely used questionnaires in the field of sexual function, which has been repeatedly used for breast cancer survivors, either alone or together with other questionnaires such as FSD[34], SQOL-FEORTC QLQ-C30[35] MBSRQ[28]. None of these instruments are specific to breast cancer. While breast cancer requires special attention for these reasons:

1- In most cultures, the breast is a symbol of femininity and sexuality, and breast cancer or its treatment may have negative effects on sexuality and femininity.

2- Following breast surgery, women report a decrease in sexual arousal from breast stimulation

3- As a result of hormonal treatments for breast cancer, women may experience decreased sexual responsiveness[36].

The Female Sexual Function Scale - Adapted for Breast Cancer (FSFI-BC) is a suitable instrument for breast cancer survivors which covers the limitations of other tools by asking questions about changes in sexual function after cancer diagnosis and treatment , examining the use of lubricants and the role of psychological distress. It’s a 34-item scale with 8 subscales including: changes after cancer, desire, arousal, lubrication, orgasm, pain, satisfaction and distress. 4 additional items were added to evaluate partner role only for clinical interpretation. 19 out of 34 items were similar in both sexually active (SA) and non- sexually active (NSA) women. The remaining 15 items separately assess lubrication, orgasm, arousal and pain in SA group and if they have difficulty in these items for NSA group. 7 factors (changes after cancer, desire/arousal, lubrication, orgasm, pain, satisfaction and distress) identified in EFA account for 79.98% (SA) and 77.19% (NSA) variance in responses. Scale also showed acceptable ICC as 0.89-0.96(SA) and 0.71-0.96(NSA).Test-retest reliabilities (SA:r=0.71-0.88, NSA: r= 0.63-0.86) were evident too. Several other measures like sexual problem scale, body image scale, fatigue assessment scale were used with the FSFI-BC to affirm convergent and divergent validity. Both SA and NSA women showed positive feedback to FSFI-BC (Bartula 2015). This tool is in English and there is no Persian version. Therfor, the main aim of this study was translation of the FSFI-BC to Persin and evaluation its psychometric properties in Iranian breast cancer survivors.

**Methods**

Present cross- sectional methodological study was conducted on female breast cancer survivors from June to November 2018**.**

**Study sample**

The sample used for this study was selected through convenience sampling method. The participants were women suffering from non-metastatic cancer in the stages of 1 or 2 who were 18 years old or more and at least 6 months had passed from their last cancer treatment. The participants were selected from the Cancer Research Center of Shahid Beheshti University of Medical Sciences. Since there was no access to enough samples, some of the data was collected from the Oncology Clinic of Shohadaye Tajrish Hospital, and the others from electronic questionnaires in the cyberspace. All participants signed the informed consent form. Verbal informed consent was obtained from people without formal education. Getting verbal consent from these participants was acceptable by ethics committee of Shahid Beheshti University of Medical Sciences (SBMU).The questionnaire items were read to them by a research assistant and their answers were recorded.

**Sample size**

There is no general consensus over sampling adequacy in psychometric studies and various instructions are provided[37].To obtain an optimal sample size , Gorsuch suggests five respondents per item, and that the sample size should not be less than 100[38]. Some have suggested 3 participants for each variable[37]. Munroe believes that a sample size of 100-200 subjects is sufficient for most purposes[39]. In this study , 100 people in each group of SA and NSA women were considered for confirmatory factor analysis.

**The study instrument (FSFI-BC)**

This tool is a self-reporting scale with 34 items (with a 5 or 6 point Likert scale) and 8 sub-items as follows: post cancer changes, desire, arousal, lubrication, orgasm, pain, satisfaction, and distress. In order to help clinical interpretation, 4 items regarding the role of husband in sexual dysfunction were added. 19 items were similar for all and 15 others separately evaluated arousal, orgasm, lubrication and pain and to see whether these problems are because of not having sexual relationship (for NSA women). Higher scores in any area indicate better sexual function. Scores of less than 15, 18, 12, 12, 9, 9 in subscales of changes in sexual function after breast cancer diagnosis and treatment, distress, desire, lubrication, orgasm and pain are considered as sexual dysfunction and require consideration[40].

**Translation**

In this study, the Wilde model proposed by Ebadi et al. (2005) was used[37]. In this way, After getting consent from Bartula, forward-backward method was used to translate FSFI-BC from English to Persian. First, the English version of the research questionnaire was translated to Persian by one of the researchers and a PhD student of reproductive health separately. Then, the translated text was read out for comparison one by one question. Where necessary, an English translator was asked for advice, until single translation was prepared.

In the next step, a sexology professor was asked to check for the fluency and the conceptual correctness of the questionnaire items. In the backwards stage, two English language experts were asked to translate the Persian version into English again. After comparing the two backward translations by the researcher and a translator, a unit English version was prepared. This version was sent to the instrument designer via Email, and she was asked to confirm the translated options with the initial questionnaire. After adjustments, this questionnaire was tested in a pilot study for 10 cases. Then, the final version was prepared and used in the study.

[38]

**Statistical analysis**

Different statistical tests were used to evaluate psychometric properties of the Persian version of FSFI as follows:

**Face and content validity**

Qualitative and quantitative methods were used for content validity. In the qualitative method, a questionnaire was given to 10 experts including 8 reproductive health specialists who were faculty members too, one psychologist and one psychiatrist. They were asked to comment on the terminology, grammar and the order of the items.

For quantitative part , CVR (content Validity Ratio) and CVI (Content Validity Index) were calculated.

According to Lawshe table, CVR more than 0.62 (according to the number of experts) and CVI of 80 percent according to Waltz and Bassel index were considered acceptable[37, 41].

For face validity, 10 breast cancer survivors who met the inclusion criteria of the study were selectedThe questions of Persian version were read to them one by one and they were asked to tell if the question was clear and understandable enough by giving a score of 1 to 5. Then, impact score was calculated.Values more than 1.5 were accepted[37].

**Construct validity**

Considering that the dimensions of the questionnaire were clearly defined, only confirmatory factor analysis (CFA) was necessary .The confirmatory indicators were acceptable, so there was no need for exploratory factor analysis(EFA). Using the EFA, only the percentage of variance explained by the factors was calculated, and the purpose was not to identify the factors, because the factors were well identified in the main questionnaire, and in our study, these factors were confirmed using CFA.

CFAand EFA were performed to determine which tool elements are correlated. In order to investigate the goodness of fit of the whole model, normalized fit index (NFI), comparative fit index (CFI), root mean square error of approximation (RMESA), and chi square (df/χ ^2^) were used. So, goodness of fit index (GFI) was over 0.8, comparative fit index was from 0.9 to 0.95, RMESA less than 0.05 showing a good fit, 0.05 to 0.08 showing an acceptable fit, from 0.08 to 0.1 showing an average fit, and over 0.1 showing weak fit; and finally, chi square of less than 3 indicated good fit between the model and the data [37, 42].

**Reliability**

Cronbach's alpha coefficient was used to determine the internal consistency of the questionnaire. The values equivalent to 0.7 or more were considered acceptable. Also, test- retest reliability was conducted with a four -week interval to determine Intra Class Correlation (ICC). The value of ICC was considered acceptable if it was 0.8 or more[37].

[43].

**Results**

**Socio-demographic and clinical status**

Altogether 200 participants were considered for this study. Some individuals were dropped of the study because they were not available to complete follow up questionnaire after four weeks. Therefore, sampling continued to reach the optimal sample size of 200 participants. The majority of them were housewives and their mean age was 48.91±9/758.The mean age at diagnosis of breast cancer was 45.3 years and mastectomy was performed for 116 (58%) individuals. For the remaining 42%, only the mass was removed. All the participants were married and had completed the initial treatment including surgery, chemotherapy and radiotherapy process. The details about participants are presented in table 1.

**Table 1 – Demographic characteristics of study participants (n=200)**

| Variable | | Mean(SD) |
| --- | --- | --- |
| Age  Age at cancer diagnosis | | 48.91(9.75)  45.3(9.48) |
| Variable | | N0.(%) |
| Job | Housewife | 148(74) |
|  | Employed | 36(18) |
|  | Retired | 36(18) |
| education | Illiterate no formal education | 10(5) |
|  | Elementary | 23(11.5) |
|  | Mid-school | 31(15.5) |
|  | Diploma | 68(34) |
|  | University grade | 68(34) |
| Surgery type | Mastectomy | 116(58) |
|  | Lumpectomy | 84(42) |

**FSFI-BC score range**

Descriptive findings of FSFI-BC score range are presented in table 2. Regarding the fact that if changes after cancer, distress, desire/arousal, lubrication, orgasm, and pain take scores of less than 15, 18, 12, and 9 respectively, they are considered as sexual dysfunctions and require follow-up.

For people who have not been sexually active during the last 4 weeks, these mentioned areas were described whether these problems have led to abstinence from sexual intercourse. The results indicate that both groups of sexually active (SA) and non-sexually active (NSA) have lower points in changes after cancer (3.537 in SA vs.8.42 ± 3.761 in NSA±10.58). But for distress, the results for both groups were higher than expected regarding sexual dysfunction.

**Face and content validity**

After completing the translation process, the face validity for items in Persian version of FSFI-BC was approved by 10 breast cancer survivors. During qualtitative face validity, no item was omitted and for quantitative face validity, The impact score of each item was determined. The lowest value of the obtained score was 3.7, which was more than the acceptable impact score of 1.5. In this way, all the items considered acceptable for further analysis.

**Content validity**

Both quantitative and qualitative methods were used for evaluation of content validity. For qualitative content validity, The questionnaire was given to 10 experts who were familiar with the research topic to provide their corrective views in the field of compliance with Persian grammar, use of appropriate words, necessity, importance, placement of expressions and scoring. The proposed amendments were reviewed by the research team and the agreed items were included. For quantitative content validity CVI and CVR were calculated for each item. Based on the number for specialists CVI value > 0.79 and CVR value>0.62 considered acceptable. The CVI of items 6 and 33 was less than 0.62. According to the research team, item

33 remained and item 6 is related to the partner, which had no effect on the overall scoring, so it was automatically removed.

**Table 2- Descriptive statistics for FSFI-BC**

| Domain | Mean(SD) NSA^*^ | Mean(SD)  SA^**^ | Mean score indicate sexual dysfunction |
| --- | --- | --- | --- |
| Changes after cancer | 8.42(3.761) | 10.58(3.53) | <15 |
| Distress | 20.82(8.845) | 22.63(6.42) | <18 |
| Desire/arousal | 10.50(4.82) | 16.15(4.42) | <12 |
| Lubrication | 7.90(4.24) | 14.82(5.21) | <12 |
| Orgasm | 6.50(3.51) | 10.29(3.14) | <9 |
| Pain | 7.55(3.91) | 10.26(3.82) | <9 |

*NSA= Non sexually active women **SA= Sexually active women

**Construct validity**

**Confirmatory factor analysis-**To evaluate the model fit, three categories of absolute fit, comparative fit and parsimonious fit are used. It is generally believed that at least one indicator should be reported from each category. In this study, NFI, CFI, RMSEA and χ 2 / df indices were reported as showed in table 3.

Optimal values for these indices were considered more than 0.9, more than 0.9, less than 0.08 and less than 2, respectively. Therefore, the validity of the construct with confirmatory factor analysis showed that the questionnaire had an acceptable validity.

**Table 3-** Values of fit indices of the confirmatory factor analysis model for

FSFI-BC questionnaire

| Fit indicators | Values in NSA group | Values in SA group |
| --- | --- | --- |
| Normed fit index(NFI) | 0.9 | 0.9 |
| Comparative fit index(CFI) | 0.92 | 0.93 |
| Root mean square error of approximation(RMSEA) | 0.075 | 0.073 |
| Chi square divided to degree of freedom(χ 2 / df) | 1.71 | 1.68 |

**Exploratory factor analysis-**

In exploratory factor analysis (EFA), KMO (Kaiser-Meyer-Olkin) index for NSA individuals was 0.786 and p <0.0001 and in total 6 instrument factors explain 74.60% of the total variance. In the SA group, the KMO index was 0.821 and p <0.0001, which explains 75.756% of the total variance (KMO value is considered 0.7-0.8).

Findings of the study showed that all items have a factor load above 0.3 in the relevant field.

**Reliability-**

The Cronbach's alpha coefficient varied from 0.818 to 0.969 indicating high internal consistency. Since an acceptable alpha is 0.7 or more, we can conclude that this questionnaire has high reliability.

**Discussion**

The present study was performed to translate and determine the psychometric properties of the FSFI-BC scale. This tool has been designed for sexual dysfunction screening in breast cancer survivors. The results of the study showed excellent psychometric properties for this scale. In FSFI-BC, 4 questions about spouse / sexual partner and 5 questions related to sexual life satisfaction were not included in the overall score. The above items were also omitted during the factor analysis stages in our study, and only items whose scores were analyzed in the tool subgroups of the Bartula and Sherman study were reviewed[40].

The results of the present study showed that the questionnaire is a valid and reliable tool for screening of sexual dysfunction in breast cancer survivors. The reliability of this scale was calculated through the internal consistency coefficient (ICC) and the reliability coefficient of test-retest. Cronbach's alpha coefficient in domains for the whole instrument ranged from 0.885 to 0.945. Also, the value of alpha coefficient for the group with sexual activity ranged from 0.811 to 0.902 and for the group without sexual activity from 0.74 to 0.949, which indicates high internal consistency. The results of this study were similar to those of Bartula and Sherman. In their study, the Cronbach's alpha coefficient varies from 0.71 to 0.96 for the group without sexual activity and from 0.89 to 0.96 for the group with sexual activity. This indicates the high consistency of the results. [40].

In the test-retest, the correlation between the two stages in the whole instrument ranged from 0.918 to 0.972, in the group with sexual activity from 0.817 to 0.974 and for the group without sexual activity from 0.974 to 0.993. This indicates high correlation in test-re- test. This high correlation was also observed in Bartula study so that the test-retest result was reported in the group without sexual activity with a correlation coefficient of 0.63 to 0.86 and in the group with sexual activity 0.71 to 0.88[40]. Internal consistency and stability of scores over time were two of the main indicators of reliability, the former can be calculated through Cronbach's alpha coefficient and the latter through test-retest [44, 45]. On the other hand, CVI and CVR also indicate the validity of good scale content.

The construct validity of the scale was analyzed by confirmatory factor analysis method and the results confirmed the 6-factor model presented by Bartula and Sherman. The results of exploratory factor analysis also showed that the 6-factor model has a good fit like the original FSFI-BC model. Both groups (with and without sexual activity) were similar, and this was in line with the original FSFI-BC version[40].

In the study of Shandiz et al. And the study of Chirani et al., the Iranian version of FSFI was used to measure sexual function in breast cancer patients. Using the Iranian version of FSFI was a positive feature of their study. [26, 46]. This instrument was translated and psychometric by Mohamadi et al.. However, this instrument had desirable psychometric properties, such as in areas of high test-retest reliability (0.73-0.86) and good to excellent internal stability (α = 0.72-9.9). The difference between the Persian version of FSFI and the main example is that in the exploratory factor analysis, the 6-factor model did not fit well with the data and the 5-factor model of sexual desire / arousal, lubrication, orgasm, pain and sexual satisfaction is more appropriate than the original 6 -factor model. However, researchers have proposed to separate the two domains of desire / arousal for clinical use[47]. Despite this, in all the mentioned studies, FSFI has been validated in the patients of midwifery and gynecology clinics in the age group of 19-54 years (with an average of 29.7 ± 3.7 years) and it’s use in the group of Breast cancer patients needs validation and verification. While the FSFI-BC used in this study was designed only for breast cancer survivors.

In other studies for evaluating sexual function in breast cancer survivors, several tools have been used. However, FSFI is the most widely used tool in various type of cancers. The results of Bartula & Sherman study screening for sexual dysfunction in women with breast cancer showed that FSFI has excellent internal stability (α = 0.83-0.96) and high correlation in test-retest (r= 0.74- 0.86). According to confirmatory factor analysis, if item 14 (the degree of emotional closeness with the sexual partner) is removed, 6 subscales of desire, arousal, lubrication, orgasm, pain and satisfaction without considering the total and separate score ( CFI = 0.97, RMSEA = 0.07, TLI = 0.96) has a good fit [40]. Of course, it should be noted that the participants in this study were only women with breast cancer who were sexually active.

The FSFI-BC questionnaire is a self-reporting tool that is completed by the client in a short period of time, although this feature may lead to participant bias and this was one of the limitations of the study. This tool can be easily used by the treatment team to screen for sexual dysfunction. A special advantage of FSFI-BC is that it assesses sexual function in both sexually active and non-sexually active women.

**Conclusion**

In general, the results of this study showed that the FSFI-BC scale is a valid and reliable tool for measuring sexual function in breast cancer patients. In this study, we tried to evaluate the validity and reliability of this questionnaire through various methods, although the use of available individuals may limit the generalization of results. It has been a short time since this tool was designed, and the current study was the first opportunity for using it, so it is suggested to study the psychometric properties of this questionnaire on different samples and in different communities. Since sexual relationship has a paired nature, we suggest investigating questions regarding the sexual partner and sexual satisfaction for the future studies. Also, since the cutoff point of the study is not determined, we suggest determining an overall score for the questionnaire to facilitate screening of breast cancer patients with sexual dysfunction.

**Abbreviations**

FSFI-BC: Sexual function index adaptation for breast cancer patients; SA: Sexually Active; NSA: Non-Sexually Active;SBMU: Shahid Beheshti University of Medical Sciences; CFA: Confirmatory Factor Analysis; EFA:Exploratory Factor Analysis; df: Degrees of freedom; SD: Standard Deviations; NFI: Normal Fit Index; CFI: Comparative Fit Index; RMSEA: The Root Mean Square Error of Approximation; GFI: Goodness of Fit Index; CVI: Content Validity Index; CVR: Content Validity Ratio

**Declarations**

**Ethics approval and consent to participate**

This article was extracted from the doctoral thesis of reproductive health of Shahid Beheshti University of Medical Sciences (SBMU). The ethics committee of Shahid Beheshti University of Medical Sciences (SBMU) approved this study with the registration number of IR.SBMU. PHNM.1395.496.All participants were obtained a written informed consent before collecting data .Only 10 individuals had no formal education and getting verbal consent from them was acceptable by ethic committee of SBMU. All methods were performed in accordance with the relevant guidelines and regulations including Declaration of Helsinki.

**Consent for publication**

Not applicable

**Availability of data and materials**

The data used during the current study are available from the corresponding
author upon reasonable request.

**Competing interests**

The authors declare that they have no competing interests

**Funding**

None.

**Authors’ contributions**

MM conceptualized and designed the study, conducted data collection, contributed to data analysis and interpretation , drafted the manuscript, wrote and reviewed the article, ZK conceptualized and designed the study, contributed in data collection, analyzing and interpretation, reviewing the prepared manuscript, ME A contributed to conceptualization and designing , F LK contributed to analyzing and interpretation of the data, MN analyzed and interpreted data and HR M contributed to collecting necessary data. All authors read and approved the final manuscript.

**Acknowledgment**

We appreciate every one who helped us with this study especially breast cancer survivors who participated as study samples , oncology clinic staff of Shohadaye Tajrish hospital and the members of breast cancer virtual campaign .

**Authors’ details**

1 Assistant professor of Reproductive health, midwifery department, Islamic Azad University, Rasht Branch, Rasht, Iran

2 Associate professor of Reproductive health, Midwifery department, Shahid Beheshti University of Medical sciences

3 Professor of Surgical Oncology, Cancer Research Center, Shahid Beheshti University of Medical Sciences, Tehran, Iran

4 Professor of Psychology, Department of Psychology, Islamic Azad University, Roudehen Branch, Tehran, Iran

5 assistant professor of biostatistics, basic science department, Shahid Beheshti University of medical sciences

6 Professor of Radiation Oncology ,Department of Radiotherapy, School of Medicine Shohada-e-Tajrish Hospital Shahid Beheshti University of Medical Sciences, Tehran,Iran

References:

1. Hubbard G, Venning C, Walker A, Scanlon K, Kyle RG: **Supportive care needs of women with breast cancer in rural Scotland**. *Supportive Care in Cancer* 2015, **23**(6):1523-1532.

2. Wang LY, Pierdomenico A, Brandt R, Lefkowitz A: **Female sexual health training for oncology providers: new applications**. *Sexual medicine* 2015, **3**(3):189-197.

3. Mamishi N: **Research in Sexual Dysfunction for Women with Breast Cancer: Statistical Fulfillment or Patient Satisfaction?** *Basic & Clinical Cancer Research* 2016, **8**(1&2):1-3.

4. Abdollahzadeh F, Moradi N, Pakpour V, Rahmani A, Zamanzadeh V, Mohammadpoorasl A, Howard F: **Un-met supportive care needs of Iranian breast cancer patients**. *Asian Pacific Journal of Cancer Prevention* 2014, **15**(9):3933-3938.

5. Garrusi B, Faezee H: **How do Iranian women with breast cancer conceptualize sex and body image?** *Sexuality and Disability* 2008, **26**(3):159-165.

6. Mahdavifar N, Pakzad R, Ghoncheh M, Pakzad I, Moudi A, Salehiniya H: **Spatial analysis of breast cancer incidence in Iran**. *Asian Pacific Journal of Cancer Prevention* 2016, **17**:59-64.

7. Harirchi I, Montazeri A, Bidokhti FZ, Mamishi N, Zendehdel K: **Sexual function in breast cancer patients: a prospective study from Iran**. *Journal of Experimental & Clinical Cancer Research* 2012, **31**(1):1-6.

8. Karabulut N, Erci B: **Sexual desire and satisfaction in sexual life affecting factors in breast cancer survivors after mastectomy**. *Journal of psychosocial oncology* 2009, **27**(3):332-343.

9. Ooi PS, Draman N, Muhamad R, Yusoff SSM, Noor NM, Haron J, Ab Hadi IS: **Sexual Dysfunction Among Women With Breast Cancer in the Northeastern Part of West Malaysia**. *Sexual Medicine* 2021, **9**(3):100351.

10. Rosman LA: **Body image and sexual functioning in breast cancer survivors**: East Carolina University; 2013.

11. Tay R, Gibney T, Antill YC: **Sexual dysfunction after breast cancer: A review of treatments and strategies**. In: *Cancer Forum: 2017*; 2017: 25-31.

12. Boehmer U, Timm A, Ozonoff A, Potter J: **Explanatory factors of sexual function in sexual minority women breast cancer survivors**. *Annals of oncology* 2012, **23**(11):2873-2878.

13. Qi A, Li Y, Sun H, Jiao H, Liu Y, Chen Y: **Incidence and risk factors of sexual dysfunction in young breast cancer survivors**. *Annals of Palliative Medicine* 2021, **10**(4):4428-4434.

14. Bartula I, Sherman KA: **The Female Sexual Functioning Index (FSFI): evaluation of acceptability, reliability, and validity in women with breast cancer**. *Supportive Care in Cancer* 2015b, **23**(9):2633-2641.

15. Ebrahimi M, Haghighat S, Mehrdad N, Olfatbakhsh A, Azin A, Najafi S: **Sexual dysfunction in breast cancer: a case-control study**. *Archives of Breast Cancer* 2015:15-20.

16. Hughes MK: **Alterations of sexual function in women with cancer**. In: *Seminars in oncology nursing: 2008*: Elsevier; 2008: 91-101.

17. Jankowska M: **Sexual functioning in young women in the context of breast cancer treatment**. *Reports of practical oncology and radiotherapy* 2013, **18**(4):193-200.

18. Oberguggenberger A, Martini C, Huber N, Fallowfield L, Hubalek M, Daniaux M, Sperner-Unterweger B, Holzner B, Sztankay M, Gamper E: **Self-reported sexual health: Breast cancer survivors compared to women from the general population–an observational study**. *BMC cancer* 2017, **17**(1):1-9.

19. Stead ML, Brown J, Fallowfield L, Selby P: **Lack of communication between healthcare professionals and women with ovarian cancer about sexual issues**. *British journal of cancer* 2003, **88**(5):666-671.

20. Vaziri S, Kashani FL: **Sexuality after breast cancer: need for guideline**. *Iranian journal of cancer prevention* 2012, **5**(1):10.

21. Almeida NGd, Britto DF, Figueiredo JV, Moreira TMM, Carvalho REFLd, Fialho AVdM: **PLISSIT model: sexual counseling for breast cancer survivors**. *Revista brasileira de enfermagem* 2019, **72**:1109-1113.

22. Masjoudi M, Keshavarz Z, Akbari ME, Lotfi Kashani F, Nasiri, Malihe, Reza MH: **Barriers to Sexual Health Communication in Breast Cancer Survivors: A Qualitative Study**. *Journal of Clinical & Diagnostic Research* 2019a, **13**(5).

23. Roshandel S, Lamyian M, Azin SA, Haghighat S, Mohammadi E: **Development and validation of a guideline on sexual and reproductive health of breast cancer survivors in Iran: a mixed methods study protocol**. *Health Research Policy and Systems* 2021, **19**(1):1-10.

24. Zangeneh S, Savabi-Esfahani M, Taleghani F, Sharbafchi MR, Salehi M: **A silence full of words: sociocultural beliefs behind the sexual health of Iranian women undergoing breast cancer treatment, a qualitative study**. *Supportive Care in Cancer* 2023, **31**(1):84.

25. Masjoudi M, Keshavarz Z, Akbari ME, Lotfi Kashani F, Nasiri M, Mirzaei H: **Barriers to Sexual Health Communication in Breast Cancer Survivors: A Qualitative Study**. *Journal of Clinical & Diagnostic Research* 2019, **13**(5).

26. Shandiz FH, Karimi FZ, Rahimi N, Abdolahi M, Anbaran ZK, Ghasemi M, Mazlom SR, Kheirabadi AN: **Investigating sexual function and affecting factors in women with breast cancer in Iran**. *Asian Pacific Journal of Cancer Prevention* 2016, **17**(7):3583-3586.

27. Masjoudi M, Keshavarz Z, Akbari ME, Kashani FL: **Evaluation of Sexual Function in Breast Cancer Survivors Using Female Sexual Function Index: A Systematic**. *Sexual dysfunction* 2019, **80**:6.

28. Bakht S, Najafi S: **Body image and sexual dysfunctions: comparison between breast cancer patients and healthy women**. *Procedia-Social and Behavioral Sciences* 2010, **5**:1493-1497.

29. Che Ya SN, Muhamad R, Mohd Zain N, Zakaria R, Ishak A, Hassan II, Ab Hadi I, Yahya MM, Low WY, Liamputtong P: **Coping strategies for sexual problems and sexual dysfunction amongst Malay women with breast cancer. A qualitative study**. *Sexual medicine* 2021, **9**(3):100336-100336.

30. Faghani S, Ghaffari F: **Effects of sexual rehabilitation using the PLISSIT model on quality of sexual life and sexual functioning in post-mastectomy breast cancer survivors**. *Asian Pacific journal of cancer prevention: APJCP* 2016, **17**(11):4845.

31. Lotfi Kashani F, Shahram V, Zeinab H: **Sexual skills training, body image and sexual function in breast cancer**. *Procedia-Social and Behavioral Sciences* 2014, **159**:288-292.

32. Farajnia S, Hosseinian S, Shahidi S, Sadeghi MS: **Codifying and examine psychometric properties of Marital Sexual Function Scale (MSFS)**. *Biannual Journal of Applied Counseling* 2014, **4**(1):85-102.

33. Bartula I, Sherman KA: **Screening for sexual dysfunction in women diagnosed with breast cancer: systematic review and recommendations**. *Breast cancer research and treatment* 2013, **141**(2):173-185.

34. Hosseini SE, Ilkhani M, Rohani C, Nasrabadi AN, Gheshlagh RG, Moini A: **Prevalence of sexual dysfunction in women with cancer: A systematic review and meta-analysis**. *International journal of reproductive biomedicine* 2022, **20**(1):1.

35. de Souza Pegorare ABG, da Rosa Silveira K, No APS, Barbosa SRM: **Assessment of female sexual function and quality of life among breast cancer survivors who underwenthormone therapy**. *Mastology* 2017, **27**(3):237-244.

36. Bartula I, Sherman KA: **Screening for sexual dysfunction in women diagnosed with breast cancer: systematic review and recommendations**. *Breast cancer research and treatment* 2013, **141**:173-185.

37. Ebadi A, Zarshenas L, Rakhshan M, Zareiyan A, Sharifnia S, Mojahedi M: **Principles of scale development in health science**. *Tehran: Jame-e-negar* 2017.

38. Gorsuch RL: **Exploratory factor analysis: Its role in item analysis**. *Journal of personality assessment* 1997, **68**(3):532-560.

39. Munro BH: **Statistical methods for health care research**, vol. 1: lippincott williams & wilkins; 2005.

40. Bartula I, Sherman KA: **Development and validation of the Female Sexual Function Index adaptation for breast cancer patients (FSFI-BC)**. *Breast cancer research and treatment* 2015a, **152**(3):477-488.

41. Lawshe C: **A quantitative approach to content validity, Personal Psychology, 28 (4), 563-575**. In*.*; 1975.

42. Plichta SB, Kelvin EA, Munro BH: **Munro's statistical methods for health care research**: Wolters Kluwer Health/Lippincott Williams & Wilkins; 2013.

43. Masjoudi M, Keshavarz Z, Nasiri M, Kashani F: **Exploration unmet sexual needs and promoting sexual function in breast cancer patients**. *Tehran: A mixed study [A dissertation presented in partial fulfillment of the requirements for the degree of Doctor of Philosophy (PhD)]: School of Midwifery And Nursing* 2019.

44. Brown JD: **The Cronbach alpha reliability estimate**. *JALT Testing & Evaluation SIG Newsletter* 2002, **6**(1).

45. Koo TK, Li MY: **A guideline of selecting and reporting intraclass correlation coefficients for reliability research**. *Journal of chiropractic medicine* 2016, **15**(2):155-163.

46. Abdollahi Chirani S, Elyasi F, Mirzaian B: **Sexual Dysfunction and its Associated Factors in Breast Cancer Patients After Mastectomy**. *Iranian Journal of Psychiatry and Behavioral Sciences* 2017, **11**(1).

47. Mohammadi K, HEYDARI M, Faghihzadeh S: **The female sexual function index (FSFI): validation of the Iranian version**. 2008.
